# Short-chain fatty acids in schizophrenia: are they affected by a depressive state?

**DOI:** 10.1192/j.eurpsy.2024.1593

**Published:** 2024-08-27

**Authors:** M. Couce-Sánchez, G. Paniagua, L. González-Blanco, A. García-Fernández, C. Martínez-Cao, C. Sabater, A. Margolles, J. Bobes, M. P. García-Portilla, P. Sáiz

**Affiliations:** ^1^AGC Salud Mental IV, SESPA; ^2^Psychiatry, University of Oviedo; ^3^G05, CIBERSAM; ^4^ISPA, Oviedo, Spain

## Abstract

**Introduction:**

Short-chain fatty acids (SCFA) are bacterial metabolites that, within microbiome-gut-brain axis, make a promising research line on etiopathology of mental diseases like schizophrenia (SZ) and major depression disorder. Besides, depressive symptoms are frequent clinical features of SZ.

**Objectives:**

- Describe fecal SCFA concentrations in SZ patients.
- Analyze differences in SCFA depending on:
- Depression.
- Clinical severity, antipsychotics and antidepressants, comorbidities (pro-inflammatory state/obesity/metabolic syndrome [MetS]), lifestyle.

**Methods:**

Cross-sectional study of 67 outpatients [mean age=43.52±12.42, range=22-67; males=40 (59.7%)] with diagnosis (DSM-5) of SZ recruited from their mental health clinics in Oviedo (Spain).

- Assessment:
- Fecal SCFA (gas chromatography;μg/mL).
- Plasmatic C-reactive protein (CPR;mg/dL).
- PANSS, Calgary Depression (CDS), International Physical Activity (IPAQ), Mediterranean Diet Adherence (MEDAS).
- Toxic habits (alcohol use/smoking/cannabis).
- Chlorpromazine equivalent doses (CPZ-ED), use of antidepressants.
- MetS (ATP-III), body mass index (BMI; kg/cm2).
- Statistics: Spearman correlation, U Mann-Whitney, ANCOVA.

**Results:**

14 patients showed clinical depression (CDS≥5). There were no differences in age or sex between groups. 36 patients (53.7%) showed systemic low-grade inflammation (CPR≥0.3mg/dL) and 32 (30.8%) MetS.Table 1 shows fecal SCFA levels by depressive state. Means (SD) are ahown.

**Table 1:** 

	CDS≤4	CDS≥5	Total	U Mann-Whitney (p-value)
Acetate	21.449(12.823)	12.911(7.189)	19.665(12.328)	221.000(0.021)
Propanoate	9.170(6.819)	6.848(6.036)	8.685(6.687)	268.500(0.114)
Butyrate	8.529(6.436)	7.875(8.232)	8.392(6.787)	320.000(0.432)
Total SCFA	39.148(23.770)	31.415(24.526)	36.742(23.549)	250.000(0.062)

Correlations were found in Age with Butyrate (r=-0.248,p=0.043) and weekly alcohol units with Propanoate (r=0.250,p=0.041) plus trend to significance with Butyrate (r=0.232,p=0.059). It also showed a trend towards statistical relation for CPZ-ED with Propanoate (r=-0.253,p=0.039) and Total SCFA (r=-0.253,p=0.039). We found no correlation in SCFA with MetS, CGI, PANSS-N, BMI, IPAQ, MEDAS and other toxic habits.

ANCOVA was performed to Acetate and Total SCFA using depression state as independent variable and Age and CPZ-ED as covariates. There was a trend towards statistical significance for Acetate (F=3.937,p=0.052,η2=0.059) whereas Total SCFA showed no difference (F=1.350,p=2.250,η2=0.021).

**Conclusions:**

There seems to be lower levels of fecal Acetate in SZ patients with depressive symptoms, considering age and antipsychotic intake. In our sample there was no relation between SFCA and clinical severity, lifestyle, comorbidities or antidepressant use.

**Disclosure of Interest:**

None Declared

